# Our London Letter

**Published:** 1893-03

**Authors:** 


### LONDON LETTER.

Following the smart visit of severe frost, the British Isles are experiencing a spell of that peculiar indefinable species of weather, of which this climate can afford a very fine sample—mist, dull skies, humidity and lack of comfort. But there is a silver-lining to these leaden clouds, and that consists of the coin of the realm which the medical men receive as their emoluments in exchange for advice and medical comforts.

A correspondence has been going on recently in a London journal, noted for its honest intentions, concerning the rapid growth of the custom of physicians preparing their own prescriptions. In many large districts this is quite unknown, and strangers settling down in such centres invariably remark with what precision the members of the medical and drug professions protect each other's interests, the members of the latter confraternity even refusing to give trivial advice from behind the counter.

The outcome of the correspondence alluded to seems to be that both the professions and the general public are greatly in favor of the prescription being prepared by hands other than those by which it was written, and the reasons assigned for this are numerous. However, no interference can have any effect, as medical men are not bound by any rules over such matters, and it will remain with the practitioners themselves what to do.

Many districts of England and Scotland are suffering keenly from smallpox, and the present epidemic has proved conclusively that vast numbers of infants are not vaccinated, and this, in spite of the stringency with which the law is enforced, as a result, a controversy is raging between those in favor and those against the law directing the inoculation of children. Quite a nice increase to the usual yearly indement is taking place as regards the family doctors, in the shape of fees in payment for vaccinating adults.

Of late much attention has been paid, in some quarters, to the different varieties of fungi, and it is said an eminent London professor and lecturer is experimenting with a view to introducing such more widely into the practice of medicine. One important feature of the investigations being instituted relates to that dread disease, leprosy; and if the authority alluded to can bring his plans to a practical issue, the good ensuing to the human race will prove of incalculable value. It is to be hoped that it is not merely a flash in the pan, as so many of these new and important so-called discoveries turn out; but from the reports just to hand, and also from the interest which is being manifested in official circles, it would seem that there is likely to be something of an important nature made known at an early day.

Nothing of an encouraging character has yet been announced in connection with the large number of experiments, etc., made by a large variety of medical men, relating to the cure or prevention of cholera. There is, of course, a certain hesitancy in making public any scheme which time alone can test, unless there are present some signs of success which savour of certainty. If any subject should be made public by the offer of a large government gratuity, nothing is more worthy of the prize than a solution of the cholera mystery; as, undoubtedly, we shall have a visit of that dire plague during the forthcoming summer, and considering that it will be an eventful year, all being well, strenuous efforts should be made to discover a plan of coping with the phantom-like malady, and thus tend to promote the peace of mind of all classes.

